# Report of a Case of Triplets

**Published:** 1895-08

**Authors:** T. W. Basinger

**Affiliations:** Petersburg, Indiana


### REPORT OF A CASE OF TRIPLETS.

BY T. W. BASINGER, M. D., PETERSBURG, INDIANA.

On the 6th of April, 1895, between the hours of 7 and 8:30 A. M., I delivered Mrs. James Marsee, Petersburg, Ind., of triplets—one male, and two females. The boy was delivered first. The first girl was the largest of the three, weighing five pounds, the boy and second girl weighing the same—four pounds each. The first and last were breech presentations. There were three ammiotic sacs, an occurrence which is said to be rare; only two placentae. The first two children delivered were nourished by one placenta. The placentæ were removed with considerable difficulty, the one supporting the last born (which was the smaller of the two) was adherent to the left side of the uterus. Mother and infants are doing well, so far, three months after delivery. The mother is 41 years old, has given birth to twelve children. She has twice been delivered of twins, in 1883 and in 1892. Only one of the four is now living; therefore she has nine living children.

